# Adverse pregnancy outcome associations among women prior to a diagnosis of systemic lupus erythematosus

**DOI:** 10.3389/flupu.2026.1806496

**Published:** 2026-06-08

**Authors:** Jessica A. English, Bethany J. Wolf, Diane L. Kamen

**Affiliations:** 1 Division of Rheumatology and Immunology, Department of Medicine, Medical University of South Carolina, Charleston, SC, United States,; 2 Division of Biostatistics, Department of Public Health Sciences, Medical University of South Carolina, Charleston, SC, United States

**Keywords:** adverse pregnancy outcomes, immune dysregulation, lupus, predisease state, pregnancy, systemic lupus erythematosus

## Abstract

**Background:**

Immune dysregulation years prior to a clinical diagnosis of systemic lupus erythematosus (SLE) may include a range of asymptomatic autoantibody positivity to clinically evident disease. The effect of this spectrum of immune dysregulation on pregnancy outcomes, including pregnancies in women prior to a diagnosis of SLE, is poorly understood. We sought to identify associations of adverse pregnancy outcomes across groups along this spectrum.

**Methods:**

Utilizing a large longitudinal cohort at a single center, we evaluated pregnancy outcomes among the following four groups: antinuclear antibody (ANA) negative controls, ANA-positive controls, pregnancies before a diagnosis of SLE, and pregnancies after a diagnosis of SLE. The pregnancy outcomes considered were live birth rate, preeclampsia, low birth weight, premature birth, spontaneous abortion, and stillbirth. Generalized estimating equation models were used to evaluate key associations and confounders.

**Results:**

We included 811 participants and 2,209 pregnancies. Of these, 198 participants were ANA-positive controls and 369 were diagnosed with SLE, with their first pregnancy occurring before diagnosis. Overall, 81.5% self-identified as Black and 31% had resided in areas with high social vulnerability. The median number of pregnancies between the groups was similar, with the majority of participants having at least one live birth. The lowest median number of pregnancies occurred in the pregnancy after SLE diagnosis group. The adverse outcome rate did not differ between the ANA-positive and ANA-negative controls. The risk of any adverse outcome was greatest in those with pregnancies after an SLE diagnosis [OR (95% CI): 3.33 (2.35, 4.71)], but was also increased in those with pregnancies before a diagnosis of SLE as compared to the ANA-positive controls [OR (95% CI): 1.78 (1.28, 2.47)]. When grouped by time prior to diagnosis, the risk of any adverse outcome remained increased in pregnancies occurring both 2–5 years or greater than 5 years prior to SLE diagnosis as compared to the ANA-positive controls [OR (95% CI): 1.64 (1.01, 2.67) and OR (95% CI): 1.65 (1.17, 2.32), respectively].

**Conclusion:**

The risk of adverse pregnancy outcomes appears to increase along the SLE spectrum, with the highest risk occurring after diagnosis. ANA positivity alone is not sufficient to impact pregnancy outcomes.

## 1 Introduction

Women with systemic lupus erythematosus (SLE) have adverse pregnancy outcomes, such as preeclampsia and preterm delivery, at rates up to five times higher than the general population (1). Black women are disproportionately at risk for both SLE and these same poor pregnancy outcomes (2–4). In SLE, delay in time to diagnosis is common and may involve many years of mild symptoms that are unrecognized (5). During this time, many women are in their childbearing years and may have suffered from adverse pregnancy outcomes.

It is also well-established that serum autoantibodies associated with conditions such as SLE are detectable several years prior to diagnosis (6). Autoimmunity from a dysregulated immune system precedes a diagnosis of SLE and is historically characterized by the appearance of a positive antinuclear antibody (ANA) test (7). This “pre-SLE state” may exist on a spectrum of immune dysregulation ranging from asymptomatic autoantibody positivity to symptoms suggestive of an SLE diagnosis (8). While the risk of adverse pregnancy outcomes in SLE is known, there are few published studies evaluating these outcomes across the SLE spectrum. Prior work at our center over a decade ago, utilizing a predominantly Black cohort, suggested that a pre-SLE disease state affecting pregnancy outcomes may exist; however, whether there are immunologic or sociodemographic etiologies of these differences was less understood (8). In addition, relatives of patients with SLE are at increased risk of developing SLE, with notable differences in immunologic characteristics (9). The interaction of pregnancy in this at-risk population has not been well-studied.

The opportunity to identify adverse pregnancy outcome associations along this spectrum of SLE exists and is a gap in the literature. Pregnant women typically have increased contact with the health system for routine monitoring and thus pregnancy may serve as an ideal time to identify signs of early disease, if contributing factors are better elucidated. Therefore, the objective of this study was to evaluate associations of pregnancy outcomes across the spectrum of SLE [including early immune dysregulation and undifferentiated connective tissue disease (UCTD)] compared to an ANA-negative control population. We hypothesize that a pre-SLE state of autoimmunity exists, contributing to adverse pregnancy outcomes prior to a diagnosis of SLE.

## 2 Materials and methods

### 2.1 Study population

Pre-existing participant data were obtained for this study from a well-characterized longitudinal cohort at a single center that has been recruiting patients and controls (related and unrelated) for over 20 years (est. 2003) at the Medical University of South Carolina (MUSC). Patients and controls were recruited as part of the Core Center for Clinical Research (CCCR), Improving Health in Rheumatic Diseases (ImHeaRD), through rheumatology clinics at our institution. A secure REDCap database was utilized. In-person interviews were conducted for demographics, pregnancy history, social, medical, and family histories and updated at follow-up visits. Controls are recruited through the “bring a friend or family member” method. These included participants who self-identified according to the five U.S. Office of Management and Budget racial and two ethnic categories. Case and control female participants from the MUSC Lupus Registry with at least one prior recorded pregnancy were included in this study. Participant pregnancies were classified into the following four patient-pregnancy groups: (1) ANA-negative controls, (2) ANA-positive controls, (3) SLE but pregnancy occurred before diagnosis, and (4) SLE but pregnancy occurred after diagnosis. An additional secondary analysis grouped pregnancies occurring before SLE diagnosis as follows: pregnancy ≥5 years prior to diagnosis, pregnancy 2–5 years prior to diagnosis, or pregnancy less than 2 years prior to diagnosis. The included SLE cases met the 2019 European League Against Rheumatism/American College of Rheumatology (EULAR/ACR) or the 2012 Systemic Lupus International Collaborating Clinics (SLICC) classification criteria for SLE (10, 11). Controls, both related and unrelated, were included if currently unaffected by SLE but could have had positive autoantibodies, rheumatoid arthritis (RA), autoimmune thyroid disease, or UCTD.

### 2.2 Data collection

Demographic, clinical (including pregnancy), and laboratory data were obtained primarily through the longitudinal database described above. The MUSC Biomedical Informatics Center and the MUSC electronic medical record were used to fill in missing pregnancy and laboratory data as cohort participants signed informed consent for access to these records at the time of enrollment. Serum autoantibodies had been measured previously for the majority of cohort participants. Given the retrospective nature, the timing of initial antibody positivity was not necessarily known relative to the first pregnancy and was assessed at the time of entry into the longitudinal cohort. The autoantibodies evaluated and considered positive if present include ANA >1:80, anti-Smith, anti-double stranded DNA (anti-dsDNA), antiphospholipid antibody (APA), and anti-Sjogren’s-syndrome-related antigen A (anti-SSA). APA positivity in this study includes ever having an above lab threshold value for lupus anticoagulant, anti-cardiolipin, or beta2-glycoprotein. A history of hypocomplementemia (low C3 and/or low C4), leukopenia, and thrombocytopenia was also evaluated. If a person ever had (either before or after pregnancy) SLE with an overlapping diagnosis of systemic sclerosis, RA, or autoimmune thyroid disease, this was recorded. Other autoimmune diseases were not included due to the overall small numbers in our cohort. Hypertension and diabetes mellitus were reported in the demographics (Table 1), if ever present (not necessarily during or before pregnancy). Adverse pregnancy outcomes of interest included decreased live birth, preeclampsia, low birth weight, premature delivery, spontaneous abortion, or stillbirth. Any adverse pregnancy outcome is a composite measure of these outcomes. Each outcome was collected through either patient-reported history, ICD-10 code, and/or review of the EMR. Low birth weight was defined as a baby born weighing ≤5 lb 8oz. Premature birth, in the original database questionnaire, was self-reported. A subset was available for electronic medical record (EMR) confirmation using delivery at less than 37 weeks to define premature birth. Both utilized definitions from the PROMISSE study (12). Additional variables include age at first pregnancy, infertility (attempting to conceive for ≥1 year without conception), number of pregnancies, self reported race, education level, smoking status, insurance status, and the Centers for Disease Control and Prevention (CDC) social vulnerability index (SVI). The SVI utilizes U.S. Census tract data to identify vulnerable communities and defines an average index value of 0.50, with values of 0.75 or greater indicating high social vulnerability (13).

### 2.3 Statistical analysis

Descriptive statistics for clinical and sociodemographic characteristics were determined at the patient level and pregnancy outcomes were reported at the pregnancy level across all participants and within patient-pregnancy type.

The primary goal of this study was to evaluate the association between pregnancy outcomes with SLE and ANA antibody status using the pregnancy type described above. Pregnancy outcomes of interest included live birth, occurrence of preeclampsia, low birth weight, premature birth, spontaneous abortion, and stillbirth. Notably, few stillbirths occurred and thus this outcome was only included for descriptive purposes. We also considered a composite outcome, namely, any poor outcome, defined as having one or more of the five poor pregnancy outcomes. Associations between different pregnancy outcomes and patient-pregnancy type and demographic and clinical variables were evaluated using a series of generalized estimating equation models (GEEs), assuming a binomial distribution and logit link. GEE models include fixed effects for pregnancy type and a random patient effect to account for correlation between pregnancies in the same participant. The models also adjusted for additional relevant covariates, including race/ethnicity (Black vs. other), smoking history, education level (high school graduate vs. less), insurance status (uninsured vs. insured), age at pregnancy, number of pregnancies, high social vulnerability index, and overlapping autoimmune disease (as defined above). Exploratory univariate GEEs examining associations between pregnancy outcome and the presence of different antibodies (anti-dsDNA, anti-Smith, APA, and anti-SSA), hypertension, or diabetes were also conducted. Model assumptions were checked graphically, and transformations were considered as needed. In the models considering low birth weight as the outcome, pregnancies that ended in spontaneous abortion were excluded when fitting the models. Low birth weight was evaluated independently of gestational age at birth. Differences between participant types were evaluated using linear contrast with primary comparisons being between ANA-positive and ANA-negative controls, patients with SLE with pregnancies before vs. after disease diagnosis, and between ANA-positive patients and patients with SLE whose pregnancy occurred before SLE diagnosis.

#### 2.3.1 Missing data

Between 0% and 17.2% of observations for the variables considered in the models were missing. Age at SLE diagnosis was unavailable for four of 471 participants with SLE and age of diagnosis was imputed as the mean age of diagnosis in the observed data. Given that age at SLE diagnosis was only relevant for those with SLE and only 0.8% missingness for this variable, the mean age was used to preserve sample size and minimize bias from exclusion. For all other variables, multiple imputation using the approximate Bayesian approach, with the chained equation developed by Jolani (2018; 14) and a random effect imputation model, was used to account for clustering by subject, with 25 imputations for any missing values prior to all analyses. Results are reported based on the pooled estimates of means and standard errors across imputations (14). The variables used in imputation include patient demographics, medical comorbidity, autoantibody status, and pregnancy variables, as detailed in the Supplementary Tables. As a sensitivity analysis, we also conducted a complete case analysis and compared the results with those from the imputed data. All the analyses were conducted in R v. 4.3.2.

## 3 Results

The final study population included 811 participants and 2,209 pregnancies.

### 3.1 Participant-level characteristics

Of the 811 participants, 142 were ANA-negative controls; 198 were ANA-positive controls; 369 were diagnosed with SLE, but their first pregnancy occurred before diagnosis; and 102 were diagnosed with SLE prior to their first pregnancy (Table 1). Age at first pregnancy was similar in the ANA-negative controls, ANA-positive controls, and those who were pregnant before SLE diagnosis, while those who were pregnant after SLE diagnosis tended to be older. The median number of pregnancies between the groups was similar, though those in the ANA-positive group had a higher median number of pregnancies compared to the other three groups. The majority of the participants self-identified as Black, with the highest proportion in the ANA-positive controls, followed by the ANA-negative controls, those who were pregnant before SLE diagnosis, and finally those who were pregnant after SLE diagnosis. A higher proportion of those with SLE, regardless of pregnancy timing, had medical insurance compared to controls. A smaller proportion of those who had their first pregnancy after SLE diagnosis were ever smokers compared to the other three groups. The highest proportion of those with a high SVI was in the pregnancy after SLE diagnosis group compared to the other three groups. The rate of ever having hypertension was highest in the two SLE groups (regardless of pregnancy timing), while the diabetes rate was lowest among those who had their first pregnancy after SLE diagnosis. The prevalence of other autoimmune diseases was lowest in the ANA-negative controls, followed by the ANA-positive controls. The proportion of those with a presence of non-ANA autoantibodies, occurrence of low complement levels, and occurrence of thrombocytopenia or leukopenia increased by increasing severity type (e.g., ANA negative < ANA positive < first pregnancy before SLE < first pregnancy after SLE).

### 3.2 Pregnancy-level characteristics

Among the pregnancies, 397 occurred in the ANA-negative controls, 641 in the ANA-positive controls, 899 in those with SLE but before diagnosis, and 272 in SLE but after diagnosis (Table 2). The median time to SLE diagnosis for those who were pregnant before SLE diagnosis was 13.3 years (IQR: 6.33–23.0). Moreover, 714 participants had a pregnancy ≥5 years before diagnosis, 117 participants had a pregnancy within 2–5 years of SLE diagnosis, and 68 participants had a pregnancy less than 2 years prior to diagnosis. The women in the before SLE diagnosis group were pregnant at a younger average age compared to the other groups. The live birth rate was lowest in the pregnancy after SLE diagnosis group, followed by the pregnancy before SLE diagnosis group. In general, the poor pregnancy outcome rate was similar in the ANA-negative and ANA-positive controls. The prevalence of adverse outcomes was higher among those with SLE, though the poor outcome rate was highest among those who were pregnant after SLE diagnosis.

### 3.3 Live birth

**FIGURE 1:**
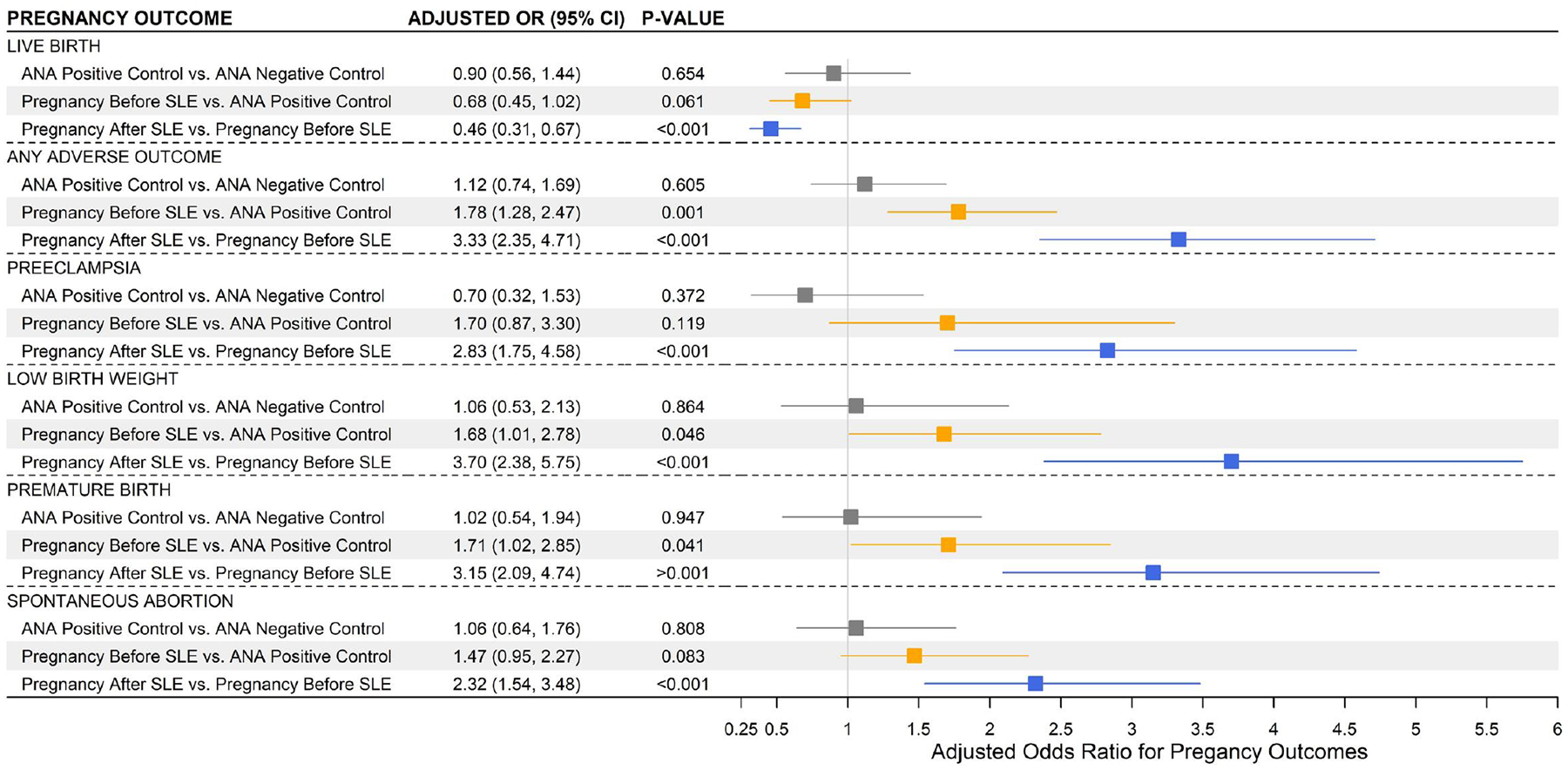
Forest plot showing the adjusted odds ratios estimated from multivariable logistic regression models for each outcome for comparisons by pregnancy type defined as follows: ANA-negative control, ANA-positive control, pregnancy before SLE diagnosis, and pregnancy after SLE diagnosis. All the models adjusted for self-reported race, smoking status, age at pregnancy, high school graduate, number of pregnancies, insurance status, social vulnerability index (high vs. low), and overlapping autoimmune disease.

In the multivariable GEE model of live birth, the occurrence of live birth was associated with pregnancy type, smoking status, number of pregnancies, education status, and occurrence of overlapping rheumatoid arthritis (Figure 1 and Supplementary Table S1). Specifically, among the women with SLE, the pregnancies that occurred after SLE diagnosis had 54% lower odds of a live birth compared to those that occurred before SLE diagnosis, after adjusting for all other factors in the model [*p* < 0.001; OR (95% CI): 0.46 (0.31, 0.67)]. The pregnancies that occurred before SLE diagnosis also had reduced odds of live birth compared to the ANA-positive control pregnancies, though this association did not achieve statistical significance [*p* = 0.061; OR (95% CI): 0.68 (0.45, 1.02)]. There was no notable difference in the odds of live birth between the ANA-positive and ANA-negative controls. In addition, being an ever smoker was associated with 46% lower odds of a live birth compared to the never smokers, after adjusting for other factors [*p* < 0.001; OR (95% CI): 0.54 (0.40, 0.72)]. An increased number of pregnancies [*p* < 0.001; OR (95% CI): 0.86 (0.79, 0.93)], being a high school graduate [*p* = 0.015; OR (95% CI): 0.52 (01, 0.88)], and having overlapping RA [*p* = 0.045; OR (95% CI): 0.67 (0.46, 0.99)] were also associated with significantly lower odds of live birth. A sensitivity analysis considering only complete cases yielded similar results, with the timing of pregnancy among those with SLE, smoking status, number of pregnancies, and education level remaining significant. However, the occurrence of overlapping RA was not significant in this sensitivity analysis.

In univariate models exploring the associations between live birth and the presence of different antibodies (anti-dsDNA, APA positivity, anti-Smith, and anti-SSA), hypertension, or diabetes, being positive for anti-dsDNA, APA, or anti-Smith was associated with lower odds of live birth. These results were consistent in the sensitivity analysis (see Supplementary Table S2).

### 3.4 Any adverse outcome

In the multivariable GEE model of any adverse outcome, the occurrence of a poor outcome was associated with pregnancy type and the presence of overlapping rheumatoid arthritis (Figure 1 and Supplementary Table S1). Specifically, among the women with SLE, pregnancies that occurred after SLE diagnosis had more than three times the odds of an adverse outcome compared to those that occurred before SLE diagnosis, after adjusting for all other factors in the model [*p* < 0.001; OR (95% CI): 3.33 (2.35, 4.71)]. The pregnancies that occurred before SLE diagnosis also had 1.8 times the odds of an adverse outcome compared to the ANA-positive control pregnancies, after adjusting for other factors [*p* = 0.001; OR (95% CI): 1.78 (1.28, 2.47)]. There was no notable difference in the odds of an adverse outcome between the ANA-positive and ANA-negative controls. Having RA, whether overlapping with SLE or in a control, was associated with significantly higher odds of an adverse pregnancy outcome [*p* = 0.029; OR (95% CI): 1.48 (1.04, 2.10)]. A sensitivity analysis considering only complete cases yielded similar results, with the timing of pregnancy among those with SLE and ANA status remaining significant. However, the association with RA was not significant in this sensitivity analysis.

In univariate models exploring the associations between any poor outcome and the presence of different antibodies (anti-dsDNA, APA positive, anti-Smith, and anti-SSA), hypertension, or diabetes, being positive for any of the antibodies, ever having low complement levels, and the occurrence of hypertension were associated with increased odds of an adverse pregnancy outcome. These results were consistent in the sensitivity analysis.

A separate multivariable GEE model of any adverse outcome was performed, wherein those with pregnancies before SLE diagnosis were subgrouped by time before diagnosis (Table 3). Pregnancies occurring both 2–5 years before and ≥5 years prior to SLE diagnosis had over 1.6 times the odds of any adverse outcome compared to the ANA-positive controls [*p* = 0.045; OR (95% CI): 1.64 (1.01, 2.67) and *p* = 0.004; OR (95% CI): 1.65 (1.17, 2.32), respectively]. Pregnancies occurring less than 2 years prior to SLE diagnosis had over 3.3 times the odds of adverse outcome compared to the ANA-positive controls [*p* < 0.001; OR (95% CI): 3.37 (1.95, 5.85)]. Pregnancies that occurred after a diagnosis of SLE had almost 3.7 times the odds of an adverse outcome compared to pregnancies that occurred either 2–5 years before or ≥5 years prior to SLE diagnosis [*p* < 0.001; OR (95% CI): 3.70 (2.25, 6.08) and *p* < 0.001; OR (95% CI): 3.68 (2.54, 5.34), respectively]. Compared to pregnancies that occurred less than 2 years before SLE diagnosis, there was a 1.8 increase in odds of adverse outcome in pregnancies that occurred after diagnosis [*p* = 0.030; OR (95% CI): 1.80 (1.06, 3.07)]. There were no differences between the ANA-positive and ANA-negative controls. RA overlap resulted in a 50% increased risk of any adverse outcome [*p* = 0.022; OR (95% CI): 1.51 (1.06, 2.14)]. Results were overall similar in the sensitivity analysis, except that three findings were no longer statistically significant: pregnancies occurring 2–5 years before SLE diagnosis versus ANA-positive controls, pregnancies occurring after SLE diagnosis versus less than 2 years before diagnosis, and RA overlap.

### 3.5 Preeclampsia

In the multivariable GEE model of preeclampsia, the occurrence of preeclampsia was only associated with pregnancy type (Figure 1 and Supplementary Table S1). Specifically, among the women with SLE, pregnancies that occurred after SLE diagnosis had 2.8 times the odds of preeclampsia compared to those that occurred before SLE diagnosis, after adjusting for all other factors in the model [*p* < 0.001; OR (95% CI): 2.83 (1.75, 4.58)]. A sensitivity analysis considering only complete cases yielded similar results, with the timing of pregnancy among those with an SLE diagnosis remaining significant.

In univariate models exploring the associations between preeclampsia and the presence of different antibodies (anti-dsDNA, APA positivity, anti-Smith, and anti-SSA), hypertension, or diabetes, being positive for anti-dsDNA and anti-Smith, history of low complement levels, and history of hypertension were associated with increased odds of preeclampsia. These results were consistent in the sensitivity analysis.

### 3.6 Low birth weight

In the multivariable GEE model of low birth weight, the occurrence of low birth weight was associated with pregnancy type and self-reported Black race (Figure 1 and Supplementary Table S1). Specifically, among the women with SLE, pregnancies that occurred after SLE diagnosis had 3.7 times the odds of low birth weight compared to those that occurred before SLE diagnosis, after adjusting for all other factors in the model [*p* < 0.001; OR (95% CI): 3.70 (2.38, 5.75)]. In addition, individuals with SLE whose pregnancy occurred before diagnosis had 1.7 times the odds of low birth weight compared to the ANA-positive individuals without a diagnosis of SLE [*p* = 0.046; OR (95% CI): 1.68 (1.01, 2.78)]. Individuals self-reporting as Black had twice the odds of having a low-birth-weight infant compared to other races, after adjusting for other factors [*p* = 0.015; OR (95% CI): 1.95 (1.14, 3.34)]. A sensitivity analysis considering only complete cases yielded similar results, although the difference between those with a pregnancy prior to SLE diagnosis and those who were ANA-positive without an SLE diagnosis was no longer significant.

In univariate models exploring the associations between low birth weight and the presence of different antibodies (anti-dsDNA, APA positivity, anti-Smith, and anti-SSA), hypertension, or diabetes, being positive for anti-dsDNA, APA, and anti-Smith; the occurrence of low complement levels; and the occurrence of hypertension were associated with increased odds of preeclampsia. These results were consistent in the sensitivity analysis.

### 3.7 Premature birth

In the multivariable GEE model of premature birth, the occurrence of this outcome was only associated with pregnancy type (Figure 1 and Supplementary Table S1). Among the women with SLE, pregnancies that occurred after SLE diagnosis had more than 3.1 times the odds of a premature birth compared to those that occurred before SLE diagnosis, after adjusting for all other factors in the model [*p* < 0.001; OR (95% CI): 3.15 (2.09, 4.74)]. The pregnancies that occurred before SLE diagnosis also had 1.7 times the odds of premature birth compared to the ANA-positive control pregnancies, after adjusting for other factors [*p* = 0.041; OR (95% CI): 1.71 (1.02, 2.85)]. There was no notable difference in the odds of premature birth between the ANA-positive and ANA-negative controls. A sensitivity analysis considering only complete cases yielded similar results, although the difference between those who were pregnant prior to SLE diagnosis and those who were ANA-positive without an SLE diagnosis was no longer significant.

In univariate models exploring the associations between premature birth and the presence of different antibodies (anti-dsDNA, APA positivity, anti-Smith, and anti-SSA), hypertension, or diabetes, being positive for anti-dsDNA and anti-Smith and the occurrence of low complement levels were associated with increased odds of premature birth. However, a diagnosis of diabetes was associated with lower odds of premature birth. These results were consistent in the sensitivity analysis.

### 3.8 Spontaneous abortion

In the multivariable GEE model of spontaneous abortion, the occurrence of spontaneous abortion was associated with pregnancy type, smoking status, number of pregnancies, and education level (Figure 1 and Supplementary Table S1). Specifically, among the women with SLE, pregnancies that occurred after SLE diagnosis had 2.3 times the odds of spontaneous abortion compared to pregnancies that occurred before SLE diagnosis, after adjusting for all other factors in the model [*p* < 0.001; OR (95% CI): 2.32 (1.54, 3.48)]. The pregnancies that occurred before SLE diagnosis also had higher odds of spontaneous abortion compared to the ANA-positive control pregnancies, though this association did not achieve statistical significance [*p* = 0.083; OR (95% CI): 1.47 (0.95, 2.27)]. Individuals who reported ever smoking had 1.9 times higher odds of having a spontaneous abortion compared to those who never smoked, after adjusting for other factors [*p* < 0.001; OR (95% CI): 1.87 (1.35, 2.59)]. An increased number of pregnancies was associated with higher odds of spontaneous abortion [*p* < 0.001; OR (95% CI): 1.19 (1.09, 1.30)]. Those who graduated high school had 2.2 times the odds of a spontaneous abortion compared to those who did not, after adjusting for other factors [*p* = 0.016; OR (95% CI): 2.18 (1.16, 4.11)]. A sensitivity analysis considering only complete cases yielded similar results; however, age at pregnancy was significant in the complete case model with a 5-year increase in age associated with a 16% increase in the odds of a spontaneous abortion [*p* = 0.039; OR (95% CI): 1.16 (1.01, 1.34)].

In univariate models exploring the associations between spontaneous abortion and the presence of different antibodies (anti-dsDNA, APA positivity, anti-Smith, and anti-SSA), hypertension, or diabetes, being positive for anti-dsDNA, APA, and anti-SSA was associated with increased odds of spontaneous abortion. These results were consistent in the sensitivity analysis, though anti-Smith positivity was significant in the complete case model but not in the imputation model.

## 4 Discussion

In this cohort of predominantly Black women either with or at-risk for SLE, we found that pregnancies were adversely affected at higher-than-expected rates, even prior to a diagnosis of SLE. Notably, the risk appears to increase as a woman moves along the SLE disease spectrum, from lupus-related preclinical autoimmunity to established disease, and ANA positivity alone is not sufficient to impact pregnancy outcomes. The risk of having any adverse outcome was highest among the women who were pregnant after a diagnosis of SLE; however, this risk was also increased among the women who were pregnant prior to SLE diagnosis compared to ANA-positive control pregnancies. Together, these findings suggest that a predisease state may exist, likely related to the development of immune dysregulation prior to the onset of clinical disease, and increases the risk of poor pregnancy outcomes.

Our findings among the ANA-positive controls are consistent with studies that concluded that ANA-positive (but otherwise unaffected) women have outcomes similar to the general population, yet a diagnosis of SLE increases the risk of adverse outcomes (15–17). Our findings support those in studies showing that in the few years leading up to a diagnosis of SLE, the risk of adverse pregnancy outcomes starts to increase (3, 18). These studies were either conducted in a non-Black population or were smaller studies evaluating a subset of pregnancy outcomes. Here, we expand the evaluation of possible adverse outcomes in a unique cohort at high risk of SLE and of adverse pregnancy outcomes in general. We demonstrated that the risk of adverse outcome was increased in our cohort more than 5 years prior to diagnosis. In addition, our study began to investigate the immunologic and demographic factors associated with these findings.

Unsurprisingly, we found that the pregnancies that occurred after diagnosis of SLE, as compared to those before SLE, were at the highest risk for adverse pregnancy outcomes across all evaluated event categories. Specifically, there were fewer live births and increased preeclampsia risk, premature births, low birth weight, and spontaneous abortions. This is consistent with prior studies that demonstrated increased risks for these adverse outcomes, often despite medical management (19–21). In a large study on electronic health records in the United States, SLE pregnancies remained complicated by adverse outcomes, especially preeclampsia and preterm births, at a rate generally unchanged over the observed time period (1989–2020) (20). Our study was unable to evaluate whether medication usage, such as hydroxychloroquine, changes these outcomes. A key limitation is that we did not have the ability to evaluate lupus disease activity during pregnancy, which is a known key characteristic of whether an SLE pregnancy results in an adverse outcome or not, as demonstrated most notably in the PROMISSE study (12). Our cohort started recruitment in 2003, and since that time, there have been several advances in knowledge and comfort level with medication safety, such as azathioprine, during pregnancy (17). There have also been advances in targeted therapies, which are sometimes used in patients with the highest risk and may have beneficial effects on pregnancy outcomes, but require further study (19). Though this is a limitation, as noted above, a study evaluating pregnancy outcomes over three decades (thus overlapping with the timeline of this study) did not find significant differences in outcomes of women with SLE over time (20). This suggests an ongoing need for evaluations of lupus disease activity and other factors that may be contributing to poor outcomes in this population despite medical management.

Our study brings to light potential opportunities for intervention, such as identifying women with SLE sooner. Early identification of lupus has been a long-standing goal and continues to be a significant challenge. Given the frequency of disease diagnosis during or around pregnancy in childbearing women, this may present a specific time in a woman’s life where medical care is more frequent, and thus an opportunity to clarify potential diagnoses in the setting of vague clinical signs or symptoms. This concept was investigated in a prospective study in Italy that evaluated the prevalence of undiagnosed rheumatic disease in the first trimester of pregnancy, with positive findings (22). It has been suggested that the immune system changes in a normal pregnancy, which may lead to the progression of autoimmunity in an at-risk woman (23). This idea is supported by our finding that, compared to ANA-positive controls alone, women who were later diagnosed with SLE had pregnancies complicated by any adverse outcome. This finding was largely driven by the composite adverse pregnancy outcome. Individual adverse outcomes, such as preterm birth or low birth weight deliveries, were variably associated and should be considered exploratory. These findings are similar to those of Spinillo et al., who prospectively identified that women who had preclinical rheumatic diseases during pregnancy had higher rates of fetal growth restriction, preeclampsia, and low birth weight compared to controls (24). A study on UCTD compared pregnancy outcomes among patients with UCTD to those with SLE (low and high disease activity), finding worsening outcomes with higher disease activity; however, there was no healthy or asymptomatic control group to ascertain whether the UCTD outcomes demonstrated increased risks (25). When predisease and UCTD groups have been compared to controls, there have been findings of adverse pregnancy outcomes (26, 27). Diagnostic delay in SLE is an important consideration in the interpretation of our findings, as the interval between symptom onset or detectable immunologic changes and formal diagnosis may introduce misclassification of disease status. Some individuals categorized as not having SLE during their index pregnancy may have had evolving or subclinical disease, particularly in the 2 years prior to diagnosis, a group whose outcomes more closely resemble the postdiagnosis population. However, we also observed an increased risk of adverse pregnancy outcomes compared to ANA-positive controls among pregnancies occurring more than 2 years prior to diagnosis. Together, these findings support the presence of a preclinical disease state that affects pregnancy outcomes and highlight the need to better identify clinical and immunologic features of predisease, subclinical, and/or undiagnosed SLE during pregnancy. When investigating other factors that contribute to adverse outcomes, we discovered that if a woman has overlapping RA, the risk of any adverse outcome was also increased. This result largely seems driven by a decrease in live births among the women with overlapping RA, which is consistent with studies that found increased rates of infertility in patients with RA (28–30). In the univariate analysis, we found that ever having lupus-associated antibodies was associated with many of the adverse outcomes and could reflect the degree of immune dysregulation present in these women. A lack of any association between antiphospholipid antibodies and preeclampsia in our study was unanticipated but may be attributable to our predominantly Black cohort, as this demographic does not have APA positivity to the same degree as other populations (31). In addition, the degree of APA positivity could not be assessed in this study, which may bias the results toward the null if many low positive values or later negative values are counted inadvertently. Ever having a diagnosis of hypertension, no matter the timing of this relative to pregnancy, was associated with adverse pregnancy outcomes of any type, and specifically preeclampsia and low birth weight. This is consistent with prior studies that found a relationship between a history of preeclampsia and either chronic hypertension during pregnancy or later development of chronic hypertension (32, 33).

An important negative finding in the study is that the ANA-positive control controls did not have increased rates of poor outcomes compared to the negative controls. This is important as this is an understudied group in the published literature and further emphasizes the need to reassure women who only have ANA positivity that their pregnancy outcomes would not be expected to differ from those who are ANA-negative (34). The possibility of ANA-positive women having increased rates of recurrent pregnancy loss compared to those who are ANA-negative is a debated topic in the literature (35). Our study likely underestimates spontaneous abortions, due to higher rates of missing data in this area; however, we did not find a relationship when comparing the ANA-positive and ANA-negative groups. While the ANA-positive group did not exclude those with UCTD, RA, or autoimmune thyroid disease, these comprised a very small proportion of the group (less than 10%); thus, we do not feel this contradicts the premise of immune dysregulation contributing to poor outcomes. Rather, it further emphasizes the need for additional study in this area with larger cohorts. This is separate from our exploratory analysis that suggests a possible effect of SLE-related antibodies on adverse outcomes.

Though a well-documented phenomenon, it was unanticipated that having a high social vulnerability index, lack of insurance, or self-identified Black race was not associated with adverse outcomes overall. Being Black was only associated with increased odds of low birth weight; however, prior studies of Black women have revealed higher rates of preeclampsia, for example, compared to white women (4, 36). Lack of access to care, which would be anticipated to be more frequent among women without insurance or with high social vulnerability, would have been expected to contribute to adverse outcomes (37). As our study could not differentiate whether high SVI or the presence of insurance was associated with each pregnancy, it is possible that the individual circumstances were different at the time of pregnancy for some women. The lack of other adverse outcomes being statistically significant in Black women may be due to the low numbers of patients self-identifying as other races represented in this cohort. The presence of smoking was overall low among the women who were diagnosed with SLE compared to other groups, but smoking, older age at time of pregnancy, and an increased number of pregnancies in general were associated with an increased risk of spontaneous abortion, which was expected (38). Given these limitations in the timing of insurance status and SVI during pregnancy and limited racial diversity, our ability to detect sociodemographic differences was constrained. Therefore, the absence of observed associations with adverse outcomes cannot be interpreted as indicating that such factors do not contribute and requires further evaluation at the time of each pregnancy, for example.

This study had several limitations, many of which are characteristic of longitudinal cohort studies in general, including missing data, though we are reassured this did not significantly affect our overall results, as demonstrated by the sensitivity analysis (Supplementary Table S2). The predisease risk can be most robustly viewed as a composite outcome, whereas specific outcomes may require a larger number of “complete case” samples to better investigate them. This could be an explanation for the loss of significance for preterm birth and low birth weight in the sensitivity analysis comparing ANA-positive controls to pregnancies before SLE. Lower statistical power in some groups could also explain the loss of significance in pregnancies occurring 2–5 years prior to diagnosis compared to ANA-positive controls, while the comparison with pregnancies that occurred greater than 5 years prior retained significance, as the latter group had more than 5 times the number of pregnancies to analyze. Autoantibody status was defined as ever positive, and the temporal relationship between seropositivity and the index pregnancy could not be fully established. As a result, we were unable to confirm whether autoantibodies preceded, coincided with, or followed pregnancy, introducing potential exposure misclassification and limiting mechanistic interpretation. While we were certain the ANA-positive controls did not have established SLE at the time of the study, the unknown timing of ANA positivity relative to pregnancy may result in misclassification between the ANA-positive and ANA-negative control groups. These limitations should be considered when interpreting immunologic inferences. Our study likely underestimates the infertility rate in these groups, as the entry criteria included those with at least one prior recorded pregnancy. There is also the possibility of recall bias, as not all the pregnancy data were confirmed through electronic medical records, so the majority of the outcomes were self-reported at the time of enrollment into the study or at subsequent follow-up visits. The cohort, while including pregnancy-related questions from inception, was not designed to monitor pregnancy, so the timing of the laboratory values did not necessarily coincide with their evaluations for and diagnosis of SLE. It does, however, provide an overview of the degree of autoantibody positivity and historical lab abnormalities in the entire group. Although we were unable to determine disease activity or medication use in known SLE pregnancies, our study does display the ongoing burden of adverse pregnancy outcomes in this population. We were unable to determine whether factors that are known to impact pregnancy, such as concurrent diabetes mellitus or hypertension, were present during the time of pregnancy. We believe this is a relevant variable to evaluate, given the known relationship between these chronic conditions and pregnancy, regardless of timing. The lack of association between preeclampsia and antiphospholipid syndrome will need further evaluation. A limitation of this study is our inability to better characterize these values, and it is possible that there is an unseen effect here, given the associations in prior literature noted previously. In the future, our cohort may be better suited to elucidate medication use and the timing of these diagnoses and laboratory values; however, at this time, these cannot be integrated. To our knowledge, this study represents one of the largest evaluations of pregnancies occurring prior to SLE diagnosis in a predominantly Black cohort. Our cohort, spanning over two decades of data, allows us to take a broad look at the potential factors affecting the pregnancies of women who are later diagnosed with lupus. The inclusion of family members at higher risk for autoimmunity, many with autoantibody positivity, provides a unique comparison group to investigate how early immune dysregulation may interact with pregnancy (9). Pregnancy has been proposed as a potential environmental trigger that may unmask or precipitate autoimmunity in predisposed individuals. Immunologic shifts in the T helper (Th1/Th2) cytokine profile during pregnancy and hormonal changes have been implicated in SLE development and flares in women known to have SLE (39, 40). Interferon pathways may also play a role, with healthy pregnancies associated with the downregulation of interferon signatures, while lupus pregnancies (especially if complicated by an adverse outcome) display higher interferon signatures and interferon-*α* levels (41, 42). Elevated interferon-*λ* levels have been observed in otherwise healthy women with preeclampsia and emerging data suggest that interferon signatures increase as a person gets closer to a clinical SLE diagnosis, with interferon-*λ* being a potential early marker (43, 44). Ultimately, it is likely a combination of pregnancy-related immune changes that contribute to a break in immune tolerance and drive transitions along the SLE spectrum.

Overall, the risk of adverse pregnancy outcomes appears to increase along the SLE spectrum, with the highest risk occurring after diagnosis. Among pregnancies prior to diagnosis, the risk appears highest in pregnancies near the time of diagnosis but remains present in the years preceding it. Importantly, ANA positivity alone is not sufficient to impact outcomes, providing reassurance for clinicians and patients. We believe this study provides further evidence of an existing predisease state that affects the outcomes of pregnancies for some women before a clinical diagnosis of SLE. Moving forward, it is vital for clinicians to recognize early signs or symptoms of SLE for earlier intervention. Future research must work toward pinpointing which symptoms and/or laboratory factors impact pregnancy outcomes before a diagnosis of SLE. We plan to further investigate these immunologic factors and their temporal relationship to pregnancy in subsequent studies.

## Supplementary Material

Supplementary tables

The Supplementary Material for this article can be found online at: https://www.frontiersin.org/articles/10.3389/flupu.2026.1806496/full#supplementary-material

## Figures and Tables

**TABLE 1:** Participant characteristics.

Variable	All *(N* = 811)	ANA-negative control (*N* = 142)	ANA-positive control (*N* = 198)	Pregnancy before SLE (*N* = 369)	Pregnancy after SLE (*N* = 102)
Age at first pregnancy, years, mean (SD)	22.39 (5.25)	22.84 (6.13)	21.64 (4.94)	21.5 (4.47)	26.42 (5.48)
Number of pregnancies, median (IQR; max)	2 (2, 3; 16)	2 (2, 3; 14)	3 (2, 4; 16)	2 (2, 3; 7)	2 (1, 2; 8)
Infertility, *n* (%)	40 (5.71)	4 (3.67)	11 (5.95)	21 (6.48)	4 (4.82)
Self-identified as Black, *n* (%)	661 (81.5)	119 (83.8)	184 (92.93)	285 (77.24)	73 (71.57)
Insured, *n* (%)	766 (94.45)	130 (91.55)	186 (93.94)	351 (95.12)	99 (97.06)
Ever smoker, *n* (%)	220 (27.99)	41 (29.93)	54 (27.98)	109 (30.36)	16 (16.49)
High social vulnerability index, *n* (%)	239 (31.04)	43 (30.94)	59 (30.57)	104 (30.06)	33 (35.87)
Ever diagnosed with hypertension, *n* (%)	408 (52.85)	41 (30.6)	90 (47.62)	229 (63.97)	48 (52.75)
Ever diagnosed with diabetes, *n* (%)	125 (16.28)	22 (16.54)	36 (19.15)	61 (17.09)	6 (6.67)
Systemic sclerosis overlap, *n* (%)	20 (2.47)	0 (0)	0 (0)	16 (4.34)	4 (3.92)
Rheumatoid arthritis overlap, *n* (%)	82 (10.11)	1 (0.7)	7 (3.54)	65 (17.62)	9 (8.82)
Autoimmune thyroid disease overlap, *n* (%)	29 (3.58)	1 (0.7)	4 (2.02)	19 (5.15)	5 (4.9)
Anti-dsDNA positive, *n* (%)	358 (47.86)	0 (0)	9 (4.69)	262 (72.58)	87 (86.14)
Anti-Smith positive, *n* (%)	207 (30.44)	0 (0)	14 (7.45)	140 (45.31)	53 (56.99)
Anti-SSA positive, *n* (%)	174 (26.17)	1 (1.11)	15 (7.94)	118 (39.73)	40 (44.94)
Anti-phospholipid Ab positive, *n* (%)	142 (20.73)	2 (2.27)	21 (11.05)	82 (26.03)	37 (40.22)
History of low complement (C3 and/or C4), *n* (%)	304 (43.62)	1 (1.49)	7 (4.27)	216 (59.18)	80 (79.21)
Thrombocytopenia, *n* (%)	77 (12.58)	0 (0)	2 (1.61)	53 (15.59)	22 (22.22)
Leukopenia, *n* (%)	172 (28.01)	3 (6.12)	12 (9.6)	118 (34.5)	39 (39.8)

**TABLE 2:** Pregnancy characteristics.

Variable	All (*N* = 2,209)	ANA-negative control (*N* = 397)	ANA-positive control *(N* = 641)	Pregnancy before SLE *(N* = 899)	Pregnancy after SLE (*N* = 272)
Age at pregnancy, years, mean (SD)	25.29 (5.93)	25.77 (6.48)	25.43 (6.23)	23.99 (5.17)	28.67 (5.48)
Live birth, *n* (%)	1,883 (85.24)	355 (89.42)	567 (88.46)	762 (84.76)	199 (73.16)
Preeclampsia, *n* (%)	156 (8.23)	18 (6.08)	22 (3.91)	61 (7.55)	55 (24.02)
Low birth weight, *n* (%)	197 (10.19)	17 (5.5)	32 (5.63)	86 (10.55)	62 (25.62)
Premature birth, *n* (%)	228 (11.18)	26 (7.26)	38 (6.48)	93 (11.07)	71 (27.73)
Spontaneous abortion, *n* (%)	287 (12.99)	38 (9.57)	65 (10.14)	116 (12.9)	68 (25)
Stillbirth, *n* (%)	39 (1.77)	4 (1.01)	9 (1.4)	21 (2.34)	5 (1.84)
Any adverse outcome, *n* (%)	691 (31.28)	88 (22.17)	140 (21.84)	298 (33.15)	165 (60.66)

**TABLE 3:** Multivariable GEE model of any adverse pregnancy outcome by disease status and time from pregnancy to SLE diagnosis, if applicable.

Disease status	Any adverse outcome OR (95% CI)	*P*-value
ANA-positive vs. ANA-negative controls	1.11 (0.73, 1.68)	0.622
Pregnancy >=5 years before SLE diagnosis vs. ANA-positive controls	**1.65** (**1.17, 2.32)**	**0.004**
Pregnancy 2–5 years Before SLE Diagnosis vs. ANA-positive controls	**1.64** (**1.01, 2.67)**	**0.045**
Pregnancy <2 years before SLE Diagnosis vs. ANA-positive controls	**3.37** (**1.95, 5.85)**	**<0.001**
Pregnancy after SLE diagnosis vs. >=5 years before diagnosis	**3.68** (**2.54, 5.34)**	**<0.001**
Pregnancy after SLE diagnosis vs. 2–5 years before diagnosis	**3.70** (**2.25, 6.08)**	**<0.001**
Pregnancy after SLE diagnosis vs. < 2 years before diagnosis	**1.80** (**1.06, 3.07)**	**0.030**
Additional variables in the multivariable model		
Black	1.36 (0.97, 1.90)	0.077
Ever smoker	1.25 (0.95, 1.63)	0.109
Age at pregnancy, 5-year increase	0.99 (0.89, 1.09)	0.778
Number of pregnancies, increases by 1	1.06 (0.99, 1.14)	0.085
Highschool graduate	1.48 (0.97, 2.26)	0.072
Uninsured	0.96 (0.58, 1.59)	0.868
High social vulnerability index	0.95 (0.73, 1.23)	0.677
Systemic sclerosis overlap	1.75 (0.86, 3.54)	0.120
Rheumatoid arthritis overlap	**1.51** (**1.06, 2.14)**	**0.022**
Autoimmune thyroid disease overlap	0.66 (0.31, 1.40)	0.274

Models were developed using multiple imputed data. Values shown in bold are statistically significant.

## Data Availability

The data analyzed in this study are subject to the following licenses/restrictions: requests from investigators to utilize cohort data are reviewed and approved by the Core Center for Clinical Research executive committee. Requests to access these datasets should be directed to https://medicine.musc.edu/center-rheumatic-diseases.
