# Supplementary material for: Adverse pregnancy outcome associations among women prior to a diagnosis of systemic lupus erythematosus
Source: Front Lupus. Author manuscript; Available in PMC 2026 Jul 16. (PMC13372250; doi:10.3389/flupu.2026.1806496)
Supplement: Supplementary tables [file NIHMS2193630-supplement-Supplementary_tables.docx]

Supplementary Material

**Supplementary Table 1**: Multivariable and univariate GEE models of different pregnancy outcomes. Models were developed on multiply imputed data.

|  | **Live Birth** | | **Any Adverse Outcome** | | **Preeclampsia** | | **Low Birth Weight** | | **Premature Birth** | | **Spontaneous Abortion** | |
| --- | --- | --- | --- | --- | --- | --- | --- | --- | --- | --- | --- | --- |
| *Multivariable Model by Disease Status*​ | **OR**  **(95% CI)** | **P** | **OR**  **(95% CI)** | **P** | **OR**  **(95% CI)** | **P** | **OR**  **(95% CI)** | **P** | **OR**  **(95% CI)** | **P** | **OR**  **(95% CI)** | **P** |
| ANA Positive Control  vs.  ANA Negative Control | 0.90  (0.56, 1.44) | 0.654 | 1.12  (0.74, 1.69) | 0.605 | 0.70  (0.32, 1.53) | 0.372 | 1.06  (0.53, 2.13) | 0.864 | 1.02  (0.54, 1.94) | 0.947 | 1.06  (0.64, 1.76) | 0.808 |
| Pregnancy After SLE  vs.  Pregnancy Before SLE | **0.46**  **(0.31, 0.67)** | **<0.001** | **3.33**  **(2.35, 4.71)** | **<0.001** | **2.83**  **(1.75, 4.58)** | **<0.001** | **3.70**  **(2.38, 5.75)** | **<0.001** | **3.15**  **(2.09, 4.74)** | **<0.001** | **2.32**  **(1.54, 3.48)** | **<0.001** |
| Pregnancy Before SLE  vs.  ANA Positive Control | 0.68  (0.45, 1.02) | 0.061 | **1.78**  **(1.28, 2.47)** | **0.001** | 1.70  (0.87, 3.30) | 0.119 | **1.68**  **(1.01, 2.78)** | **0.046** | **1.71**  **(1.02, 2.85)** | **0.041** | 1.47  (0.95, 2.27) | 0.083 |
| *Additional Variables in Multivariable model* |  |  |  |  |  |  |  |  |  |  |  |  |
| Black | 0.98  (0.67, 1.43) | 0.904 | 1.37  (0.98, 1.92) | 0.067 | 1.00  (0.60, 1.67) | 0.993 | **1.95**  **(1.14, 3.34)** | **0.015** | 1.22  (0.72, 2.05) | 0.455 | 0.96  (0.64, 1.45) | 0.859 |
| Ever smoker | **0.54**  **(0.40, 0.72)** | **<0.001** | 1.22  (0.94, 1.6) | 0.140 | 0.62  (0.35, 1.1) | 0.102 | 1.36  (0.90, 2.04) | 0.143 | 0.72  (0.48, 1.1) | 0.131 | **1.87**  **(1.35, 2.59)** | **<0.001** |
| Age at Pregnancy,  5-year increase | 0.93  (0.82, 1.06) | 0.261 | 1.00  (0.90, 1.10) | 0.983 | 1.09  (0.91, 1.31) | 0.357 | 0.97  (0.83, 1.13) | 0.675 | 0.92  (0.80, 1.06) | 0.229 | 1.13  (0.99, 1.05) | 0.079 |
| Pregnancy Number,  increase by 1 | **0.86**  **(0.79, 0.93)** | **<0.001** | 1.06  (0.99, 1.13) | 0.116 | 0.93  (0.81, 1.07) | 0.303 | 0.92  (0.81, 1.04) | 0.180 | 0.94  (0.83, 1.06) | 0.290 | **1.19**  **(1.09, 1.30)** | **<0.001** |
| Highschool Graduate | **0.52**  **(0.31, 0.88)** | **0.015** | 1.53  (1.00, 2.33) | 0.051 | 1.04  (0.48, 2.24) | 0.923 | 1.08  (0.57, 2.04) | 0.810 | 1.15  (0.61, 2.18) | 0.670 | **2.18**  **(1.16, 4.11)** | **0.016** |
| Uninsured | 1.17  (0.66, 2.08) | 0.584 | 0.98  (0.59, 1.63) | 0.936 | 1.33  (0.53, 3.34) | 0.547 | 0.74  (0.30, 1.86) | 0.523 | 0.75  (0.36, 1.58) | 0.449 | 0.64  (0.32, 1.29) | 0.215 |
| High Social Vulnerability  Index | 1.16  (0.85, 1.60) | 0.346 | 0.97  (0.75, 1.26) | 0.818 | 1.10  (0.70, 1.74) | 0.673 | 1.14  (0.79, 1.64) | 0.488 | 1.12  (0.77, 1.62) | 0.546 | 0.91  (0.65, 1.27) | 0.585 |
| Systemic Sclerosis  Overlap | 0.52  (0.24, 1.12) | 0.095 | 1.76  (0.84, 3.69) | 0.132 | 1.85  (0.72, 4.72) | 0.200 | 1.55  (0.61, 3.96) | 0.360 | 0.94  (0.36, 2.47) | 0.897 | 1.87  (0.85, 4.10) | 0.117 |
| Rheumatoid Arthritis  Overlap | **0.67**  **(0.46, 0.99)** | **0.045** | **1.48**  **(1.04, 2.10)** | **0.029** | 0.96  (0.50, 1.85) | 0.907 | 1.44  (0.87, 2.40) | 0.156 | 1.01  (0.59, 1.74) | 0.963 | 1.31  (0.85, 2.01) | 0.227 |
| Autoimmune Thyroid  Disease Overlap | 1.30  (0.66, 2.57) | 0.453 | 0.63  (0.30, 1.33) | 0.228 | 0.68  (0.15, 3.05) | 0.619 | 0.59  (0.23, 1.50) | 0.270 | 0.71  (0.19, 2.68) | 0.618 | 0.60  (0.26, 1.42) | 0.246 |
| *Univariate analysis only*​ |  |  |  |  |  |  |  |  |  |  |  |  |
| anti-dsDNA positive | **0.58**  **(0.44, 0.78)** | **<0.001** | **2.13**  **(1.68, 2.69)** | **<0.001** | **2.15**  **(1.44, 3.2)** | **<0.001** | **2.16**  **(1.53, 3.04)** | **<0.001** | **1.90**  **(1.35, 2.67)** | **<0.001** | **1.68**  **(1.23, 2.29)** | **0.001** |
| anti-APA positive | **0.64**  **(0.45, 0.91)** | **0.013** | **1.54**  **(1.17, 2.04)** | **0.002** | 1.39  (0.88, 2.2) | 0.156 | **1.61**  **(1.05, 2.45)** | **0.028** | 1.40  (0.91, 2.16) | 0.129 | **1.58**  **(1.10, 2.29)** | **0.014** |
| anti-Smith positive | 0.75  (0.54, 1.04) | 0.086 | **1.69**  **(1.29, 2.22)** | **<0.001** | **1.92**  **(1.30, 2.85)** | **0.001** | **1.87**  **(1.28, 2.72)** | **0.001** | **1.56**  **(1.10, 2.21)** | **0.013** | **1.43**  **(1.01, 2.03)** | **0.041** |
| anti-SSA positive | **0.68**  **(0.49, 0.94)** | **0.018** | **1.45**  **(1.10, 1.92)** | **0.008** | 1.38  (0.88, 2.17) | 0.160 | 1.30  (0.87, 1.93) | 0.195 | 1.24  (0.84, 1.82) | 0.275 | **1.44**  **(1.02, 2.06)** | **0.041** |
| History Low Complement | 0.76  (0.56, 1.02) | 0.067 | **1.76**  **(1.39, 2.23)** | **<0.001** | **1.66**  **(1.12, 2.46)** | **0.011** | **2.14**  **(1.5, 3.05)** | **<0.001** | **1.94**  **(1.36, 2.77)** | **<0.001** | 1.32  (0.95, 1.84) | 0.099 |
| Ever Hypertension | 0.87  (0.65, 1.16) | 0.334 | **1.44**  **(1.14, 1.82)** | **0.002** | **1.60**  **(1.06, 2.41)** | **0.026** | **1.51**  **(1.04, 2.19)** | **0.030** | 1.10  (0.78, 1.56) | 0.575 | 1.10  (0.81, 1.51) | 0.540 |
| Ever Diabetes | 0.93  (0.64, 1.36) | 0.717 | 0.91  (0.67, 1.25) | 0.576 | 0.97  (0.55, 1.71) | 0.911 | 0.83  (0.50, 1.38) | 0.465 | **0.60**  **(0.36, 0.98)** | **0.043** | 0.98  (0.65, 1.48) | 0.925 |

Supplementary Table 2. Sensitivity analysis. Results from complete-case multivariable generalized estimating equation (GEE) models restricted to pregnancies with complete data. No imputation was performed.

|  | **Live Birth** | | **Any Adverse Outcome** | | **Preeclampsia** | | **Low Birth Weight** | | **Premature Birth** | | **Spontaneous Abortion** | |
| --- | --- | --- | --- | --- | --- | --- | --- | --- | --- | --- | --- | --- |
| *Multivariable Model by Disease Status*​ | **OR**  **(95% CI)** | **P** | **OR**  **(95% CI)** | **P** | **OR**  **(95% CI)** | **P** | **OR**  **(95% CI)** | **P** | **OR**  **(95% CI)** | **P** | **OR**  **(95% CI)** | **P** |
| **Disease Status** |  |  |  |  |  |  |  |  |  |  |  |  |
| ANA Positive vs. ANA Negative Control | 0.96  (0.58, 1.57) | 0.865 | 1.11  (0.72, 1.71) | 0.636 | 0.76  (0.30, 1.94) | 0.573 | 1.20  (0.53, 2.75) | 0.658 | 1.06  (0.52, 2.16) | 0.881 | 1.07  (0.63, 1.82) | 0.796 |
| Pregnancy After vs. Before SLE | **0.46**  **(0.30, 0.70)** | **<0.001** | **3.21**  **(2.19, 4.70)** | **<0.001** | **3.16**  **(1.85, 5.41)** | **<0.001** | **4.52**  **(2.75, 7.43)** | **<0.001** | **3.28**  **(2.09, 5.14)** | **<0.001** | **2.33**  **(1.50, 3.62)** | **<0.001** |
| Pregnancy Before SLE vs. ANA Positive Control | 0.67  (0.43, 1.03) | 0.069 | **1.70**  **(1.2, 2.42)** | **0.003** | 1.58  (0.79, 3.19) | 0.199 | 1.70  (0.96, 3.01) | 0.071 | 1.68  (0.96, 2.95) | 0.068 | 1.37  (0.86, 2.19) | 0.189 |
| *Additional Variables in Multivariable model* |  |  |  |  |  |  |  |  |  |  |  |  |
| Self-Identified Black | 1.08  (0.70, 1.65) | 0.738 | 1.15  (0.79, 1.68) | 0.468 | 0.92  (0.51, 1.65) | 0.781 | **1.84**  **(1.03, 3.28)** | **0.040** | 1.07  (0.60, 1.91) | 0.816 | 0.87  (0.55, 1.38) | 0.563 |
| Ever Smoker | **0.50**  **(0.36, 0.68)** | **<0.001** | 1.31  (0.98, 1.74) | 0.069 | 0.59  (0.32, 1.09) | 0.093 | 1.43  (0.92, 2.23) | 0.107 | 0.63  (0.39, 1.01) | 0.056 | **2.00**  **(1.42, 2.81)** | **<0.001** |
| Age at Pregnancy,  5-year increase | 0.91  (0.79, 1.05) | 0.182 | 1.02  (0.92, 1.13) | 0.745 | 1.08  (0.88, 1.34) | 0.464 | 0.96  (0.79, 1.16) | 0.655 | 0.91  (0.79, 1.06) | 0.230 | **1.16**  **(1.01, 1.34)** | **0.039** |
| Pregnancy Number,  increase by 1 | **0.86**  **(0.79, 0.93)** | **<0.001** | 1.05  (0.98, 1.13) | 0.197 | 0.89  (0.75, 1.05) | 0.157 | 0.90  (0.78, 1.04) | 0.163 | 0.94  (0.83, 1.07) | 0.374 | **1.17**  **(1.08, 1.28)** | **<0.001** |
| Highschool Graduate | **0.54**  **(0.32, 0.92)** | **0.024** | 1.48  (0.97, 2.25) | 0.072 | 0.97  (0.42, 2.23) | 0.949 | 1.13  (0.58, 2.17) | 0.722 | 1.14  (0.57, 2.28) | 0.720 | **2.07**  **(1.08, 3.95)** | **0.028** |
| Uninsured | 1.22  (0.68, 2.18) | 0.508 | 0.89  (0.52, 1.52) | 0.666 | 1.33  (0.48, 3.71) | 0.585 | 0.75  (0.28, 2.02) | 0.565 | 0.65  (0.30, 1.41) | 0.274 | 0.68  (0.34, 1.37) | 0.278 |
| High Social Vulnerability  Index | 1.13  (0.82, 1.55) | 0.461 | 0.95  (0.73, 1.25) | 0.724 | 1.12  (0.69, 1.81) | 0.647 | 1.11  (0.74, 1.65) | 0.610 | 1.05  (0.70, 1.58) | 0.801 | 0.92  (0.66, 1.28) | 0.606 |
| Systemic Sclerosis  overlap | 0.52  (0.25, 1.11) | 0.093 | 1.60  (0.78, 3.28) | 0.202 | 1.58  (0.59, 4.20) | 0.363 | 1.52  (0.61, 3.82) | 0.368 | 0.95  (0.36, 2.52) | 0.924 | 1.92  (0.88, 4.17) | 0.101 |
| Rheumatoid Arthritis  overlap | 0.78  (0.51, 1.19) | 0.251 | 1.39  (0.96, 2.02) | 0.079 | 1.08  (0.54, 2.16) | 0.825 | 1.60  (0.93, 2.75) | 0.090 | 1.00  (0.56, 1.80) | 0.994 | 1.23  (0.77, 1.96) | 0.389 |
| Autoimmune Thyroid  Disease overlap | 1.04  (0.50, 2.15) | 0.913 | 0.75  (0.33, 1.71) | 0.493 | 0.80  (0.17, 3.90) | 0.787 | 0.51  (0.18, 1.46) | 0.211 | 0.75  (0.16, 3.57) | 0.720 | 0.70  (0.27, 1.81) | 0.463 |
| *Univariate analysis only*​ |  |  |  |  |  |  |  |  |  |  |  |  |
| anti-dsDNA positive | **0.59**  **(0.44, 0.78)** | **<0.001** | **2.21**  **(1.73, 2.81)** | **<0.001** | **2.52**  **(1.62, 3.91)** | **<0.001** | **2.42**  **(1.66, 3.52)** | **<0.001** | **2.09**  **(1.45, 2.99)** | **<0.001** | **1.68**  **(1.23, 2.29)** | **0.001** |
| anti-APA positive | **0.64**  **(0.45, 0.92)** | **0.016** | **1.59**  **(1.18, 2.15)** | **0.003** | 1.59  (0.99, 2.54) | 0.056 | **1.74**  **(1.11, 2.73)** | **0.015** | 1.45  (0.93, 2.28) | 0.105 | **1.58**  **(1.08, 2.31)** | **0.018** |
| anti-Smith positive | 0.75  (0.54, 1.03) | 0.075 | **1.76**  **(1.35, 2.30)** | **<0.001** | **2.21**  **(1.43, 3.42)** | **<0.001** | **2.06**  **(1.40, 3.04)** | **<0.001** | **1.68**  **(1.14, 2.46)** | **0.008** | **1.43**  **(1.02, 2.01)** | **0.039** |
| anti-SSA positive | **0.68**  **(0.48, 0.94)** | **0.020** | **1.52**  **(1.16, 2.00)** | **0.003** | 1.53  (0.96, 2.45) | 0.071 | 1.36  (0.91, 2.04) | 0.136 | 1.32  (0.89, 1.96) | 0.167 | **1.46**  **(1.02, 2.01)** | **0.039** |
| History Low Complement  (C3 and/or C4) | 0.77  (0.57, 1.05) | 0.101 | **1.84**  **(1.43, 2.35)** | **<0.001** | **1.90**  **(1.24, 2.91)** | **0.003** | **2.51**  **(1.71, 3.69)** | **<0.001** | **2.22**  **(1.53, 3.22)** | **<0.001** | 1.29  (0.93, 1.80) | 0.129 |
| Ever Hypertension | 0.86  (0.65, 1.15) | 0.321 | **1.45**  **(1.14, 1.84)** | **0.002** | **1.77**  **(1.13, 2.75)** | **0.012** | **1.61**  **(1.09, 2.39)** | **0.016** | 1.11  (0.78, 1.59) | 0.548 | 1.11  (0.81, 1.51) | 0.527 |
| Ever Diabetes | 0.93  (0.64, 1.35) | 0.694 | 0.89  (0.65, 1.22) | 0.476 | 0.90  (0.49, 1.66) | 0.739 | 0.76  (0.46, 1.26) | 0.285 | **0.56**  **(0.34, 0.92)** | **0.024** | 0.98  (0.65, 1.49) | 0.935 |

**Supplementary Table 3.** Sensitivity analysis. Multivariable GEE model of Any Adverse Pregnancy Outcome by disease status and time from pregnancy to SLE diagnosis, if applicable. Complete cases only, no imputation was performed.

| **Disease Status** | **Any Adverse Outcome**  **OR (95% CI)** | **P-value** |
| --- | --- | --- |
| ANA Positive vs. ANA Negative Control | 1.11 (0.72, 1.70) | 0.645 |
| Pregnancy >=5 years Before SLE Diagnosis vs. ANA Positive Control | **1.59 (1.1, 2.29)** | **0.014** |
| Pregnancy 2-5 years Before SLE Diagnosis vs. ANA Positive Control | 1.55 (0.92, 2.63) | 0.099 |
| Pregnancy <2 years Before SLE Diagnosis vs. ANA Positive Control | **3.15 (1.73, 5.71)** | **<0.001** |
| Pregnancy After SLE Diagnosis vs. >=5 years Before Diagnosis | **3.50 (2.34, 5.25)** | **<0.001** |
| Pregnancy After SLE Diagnosis vs. 2-5 years Before Diagnosis | **3.58 (2.06, 6.20)** | **<0.001** |
| Pregnancy After SLE Diagnosis vs. <2 years Before Diagnosis | 1.77 (0.99, 3.17) | 0.055 |
| **Additional Variables in Multivariable Model** |  |  |
| Black | 1.14 (0.78, 1.66) | 0.503 |
| Ever smoker | 1.32 (0.99, 1.77) | 0.059 |
| Age at Pregnancy, 5-year increase | 1.01 (0.91, 1.12) | 0.875 |
| Pregnancy number, increase by 1 | 1.05 (0.98, 1.13) | 0.156 |
| Highschool graduate | 1.47 (0.97, 2.25) | 0.073 |
| Uninsured | 0.88 (0.51, 1.51) | 0.640 |
| High Social Vulnerability Index | 0.96 (0.73, 1.25) | 0.753 |
| Systemic Sclerosis overlap | 1.61 (0.80, 3.25) | 0.182 |
| Rheumatoid arthritis overlap | 1.41 (0.97, 2.05) | 0.069 |
| Autoimmune thyroid disease overlap | 0.78 (0.34, 1.79) | 0.558 |
